# Genotype/phenotype correlations in AARS-related neuropathy in a cohort of patients from the United Kingdom and Ireland

**DOI:** 10.1007/s00415-015-7778-4

**Published:** 2015-06-02

**Authors:** Boglarka Bansagi, Thalia Antoniadi, Sarah Burton-Jones, Sinead M. Murphy, John McHugh, Michael Alexander, Richard Wells, Joanna Davies, David Hilton-Jones, Hanns Lochmüller, Patrick Chinnery, Rita Horvath

**Affiliations:** John Walton Muscular Dystrophy Research Centre, MRC Centre for Neuromuscular Diseases, Institute of Genetic Medicine, Newcastle University, Newcastle upon Tyne, UK; Bristol Genetics Laboratory, Pathology Sciences, North Bristol NHS Trust, Southmead Hospital, Bristol, UK; Department of Neurology, Adelaide and Meath Hospitals Incorporating the National Children’s Hospital, Tallaght, Dublin, 24 Ireland; Academic Unit of Neurology, Trinity College, Dublin, Ireland; Department of Neurophysiology, Adelaide and Meath Hospitals Incorporating the National Children’s Hospital, Tallaght, Dublin, 24 Ireland; Department of Neurology, West Wing, John Radcliffe Hospital, Oxford, UK; Wellcome Trust Centre for Mitochondrial Research, Institute of Genetic Medicine, Newcastle University, Newcastle upon Tyne, UK

**Keywords:** Charcot–Marie–Tooth disease (CMT), Axonal neuropathy, Aminoacyl-tRNA synthetases (ARS), Alanyl-tRNA synthetase (AARS)

## Abstract

Charcot–Marie–Tooth disease (CMT) is the most common inherited neuropathy with heterogeneous clinical presentation and genetic background. The axonal form (CMT2) is characterised by decreased action potentials indicating primary axonal damage. The underlying pathology involves axonal degeneration which is supposed to be related to axonal protein dysfunction caused by various gene mutations. The overlapping clinical manifestation of CMT2 with distal hereditary motor neuropathy (dHMN) and intermediate CMT causes further diagnostic difficulties. Aminoacyl-tRNA synthetases have been implicated in the pathomechanism of CMT2. They have an essential role in protein translation by attaching amino acids to their cognate tRNAs. To date six families have been reported worldwide with dominant missense alanyl-tRNA synthetase (*AARS)* mutations leading to clinically heterogeneous axonal neuropathies. The pathomechanism of some variants could be explained by impaired amino acylation activity while other variants implicating an editing defect need to be further investigated. Here, we report a cohort of six additional families originating from the United Kingdom and Ireland with dominant *AARS*-related neuropathies. The phenotypic manifestation was distal lower limb predominant sensorimotor neuropathy but upper limb impairment with split hand deformity occasionally associated. Nerve conduction studies revealed significant demyelination accompanying the axonal lesion in motor and sensory nerves. Five families have the c.986G>A, p.(Arg329His) variant, further supporting that this is a recurrent loss of function variant. The sixth family, of Irish origin, had a novel missense variant, c.2063A>G, p.(Glu688Gly). We discuss our findings and the associated phenotypic heterogeneity in these families, which expands the clinical spectrum of AARS-related neuropathies.

**Electronic supplementary material:**

The online version of this article (doi:10.1007/s00415-015-7778-4) contains supplementary material, which is available to authorized users.

## Introduction

Charcot–Marie–Tooth disease (CMT) is a clinically and genetically heterogeneous group of peripheral neuropathies characterised by symmetric atrophy and weakness in the distal muscles, areflexia and variable range of sensory impairment. In respect of its worldwide prevalence of an estimated 1:2500, CMT is considered to be the most common cause of inherited peripheral neuropathies. Based on the electrophysiological and pathological characteristics it can be classified further into a demyelinating form (CMT1), where the nerve conduction velocity is reduced due to altered myelination, and to an axonal form (CMT2), where the primary axonal dysfunction leads to reduced amplitudes of evoked muscle action potentials. The intermediate form (CMTI) shares both features [1, 11, 13]. There have been more than 90 genes associated with CMT so far and, despite the overlapping phenotypes, some certain genes manifest with distinctive forms dependent on their contribution to Schwann cell and axonal functions [3].

Genetic diagnosis is particularly difficult to achieve in the axonal form (CMT2) which shows genotypic and phenotypic overlap with distal hereditary motor neuropathies (dHMN) or distal spinal muscular atrophy (dSMA), a subgroup of rare inherited motor axonal neuropathies with no significant sensory involvement [9]. Similarly, dominant intermediate CMT (DI-CMT) cases presenting with intermediate nerve conduction velocities of 25–45 m/s are also frequently misdiagnosed as CMT2 [6]. There have been 15 genes or loci described in relation to CMT2 [5]. Some of these genes have been already associated with axonal dysfunction, such as mitofusin 2 (*MFN2*) which is responsible for 5–10 % of CMT2 cases, neurofilament light chain (*NEFL*) in 1–5 % and ganglioside-induced differentiation-associated protein (*GDAP1*) in less than 1 % of the cases [1, 3, 4, 11, 13].

Aminoacyl-tRNA synthetases (ARS) are essential enzymes in the translation of the genetic code by attaching amino acids to their cognate tRNAs during protein synthesis [3, 5, 7]. There are 37 nuclear genes encoding ARSs for cytoplasmic or mitochondrial protein synthesis. Mutations in six genes encoding aminoacyl-tRNA synthetases (*ARS*) have been implicated in axonal pathology [3]. The majority of the mutations were described in glycyl-tRNA synthetase (*GARS*, MIM#600287) causing CMT2 type D (CMT2D) or distal spinal muscular atrophy type V (dSMA-V), both are autosomal dominant upper limb predominant motor axonal neuropathies. Mutations in tyrosyl-tRNA synthetase (*YARS*, MIM#603623) were reported in dominant intermediate CMT type C (CMTDIC). The relevance of methionyl-tRNA synthetase (MARS, MIM#156560) and histidyl-tRNA synthetase (HARS, MIM#142810) mutations for CMT is supported by strong functional and evolutionary evidence, yet incomplete segregation and absence of additional unrelated cases warrant future studies to substantiate this conclusion [2, 14]. Compound heterozygous lysyl-tRNA synthetase (*KARS*, MIM#601421) mutations were present in one patient with recessive intermediate CMT type B (CMTIRB) manifesting as part of a more complex neurological condition [3, 7, 15].

To date only six families have been described in the literature with dominant missense mutations in the alanyl-tRNA synthetase (*AARS*, MIM#601065) leading to clinically heterogeneous phenotypes (Table 1). The c.986G>A, p.(Arg329His) variant was reported as a recurrent mutation in two unrelated French families presenting with variable age of onset distal sensorimotor degeneration secondary to predominant axonal neuropathy and with absent or slight demyelination [5]. The same *AARS* variant was identified in a large Australian family with early onset axonal neuropathy, variable sensorineural deafness and foot deformities, accompanied by intermediate nerve conduction velocities [7]. Another Australian family with the c.2333A>C, p.(Glu778Ala) variant manifested as sensorimotor axonal neuropathy with rippling muscles and cramps in the proband, while only rippling muscles were present in three affected relatives [7]. In a Taiwanese pedigree pure axonal neuropathy with variable age of onset was associated with the c.211A>T, p.(Asn71Tyr) *AARS* variant [6]. In a three generation dominant Chinese family the c.2677G>A, p.(Asp893Asn) mutation was related to a distal motor neuropathy (dHMN) phenotype based on neurogenic EMG findings [15]. Additionally autosomal recessive loss of function *AARS* mutations (compound heterozygous p.Lys81Thr and p.Arg751Gly and homozygous p.Arg751Gly) were also described in two unrelated families, causing severe infantile epileptic encephalopathy with a central myelin defect and peripheral neuropathy [12].

**Table 1:** Summary of the clinical and electrophysiology findings accompanying the reported different *AARS* variants

Origin	Family/patient number	Nucleotide change	Amino acid change	Age onset	First sign	Clinical symptoms			Nerve conduction study		References
						Lower limb	Upper limb	Symmetry	Motor NCV (m/s)	Motor/sensory	
Taiwanese	F1/P5	c.211A>T	p.Asn71Tyr	Varied (11–30 years)	LL	Distal weakness and wasting, mild sensory loss	Mild weakness, wasting mild sensory loss	Symmetric	m 38.1 p absent	MS	[5]
	F1/P2				LL	Mild weakness and wasting	None	Symmetric			
French	F1/P16	c.986G>A	p.Arg329His	Varied (6–54 years)	LL	Bilateral distal weakness, distal sensory loss	Distal weakness, distal sensory loss	Symmetric	m 32.4–50	MS	[4]
	F1/P1				LL	Severe distal wasting, sensory loss	None	Asymmetric	m 45		
French	F2/P1	c.986G>A	p.Arg329His	14 years	LL	Mild distal weakness	None	Symmetric	m 35–39	MS	[4]
Australian	F1/P9	c.986G>A	p.Arg329His	Early	LL	Distal weakness, feet deformities, sensorineural deafness	None	Symmetric	Intermediate	MS	[2]
Australian	F1/P4	c.2333A>C	p.Glu778Ala	n/a	LL	Rippling muscles and cramps, distal wasting, mild distal sensory loss	None	Symmetric	n/a	MS	[2]
Chinese	F1/P4	c.2677G>A	p.Asp893Asn	Varied (11–55 years)	LL	Distal weakness and wasting, feet deformities	None	Symmetric	Normal	M	[6]
Mixed European	F1/P2	c.242A>C c.2251A>G	p.Lys81Yhr p.Arg751Gly	Birth/months	Generalised	Congenital vertical tali, loss of reflexes, dystonia	Dystonia	Symmetric	–	–	[12]
	F2/P1	c.2251A>G	p.Arg751Gly								
Belgian	F1/P5	c.304G>C	p.Gly102Arg	–	LL	Mild axonal neuropathy, hyperreflexia	–	–	–	–	[8]

*F* family, *P* patient, *LL* lower limb, *n/a* not available, *m/s* metre per second, *m* median nerve, *p* peroneal nerve, *M* pure motor, *MS* motor and sensory, *–* no data

Here, we report a cohort of multigenerational British and Irish families with autosomal dominant neuropathies and missense mutations in *AARS*, with the aim of expanding the clinical spectrum and providing further pheno-genotypic correlations in AARS-related neuropathies.

## Methods

### Patients

**Fig. 1:**
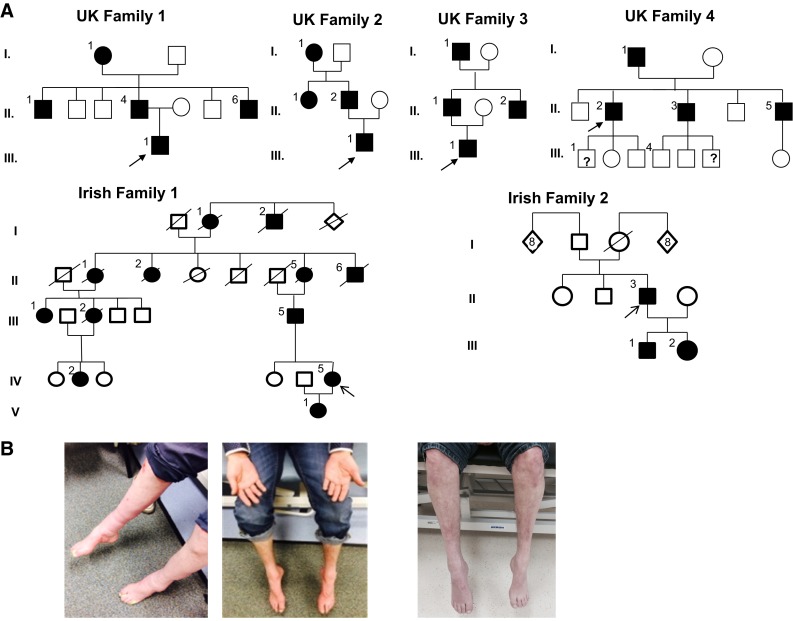
**a** Pedigrees of UK and Irish families. A*rrows* the index patients of each family. **b** Image of patient II/4 in UK family 1 showing predominantly lower limb symptoms manifesting with bilateral *pes cavus* and severe feet drop. Index patient of the same UK1 family (III/1) representing moderate intrinsic hand muscles wasting accompanied by lower limb distal wasting and weakness. Image of patient (II.1) from UK family 3 showing severe bilateral foot drop and distal muscle wasting

Clinical description and examination findings of the affected patients from 4 UK and 2 Irish families diagnosed with AARS-related neuropathy are provided in detail in Table 1 and Fig. 1 and in the Supplementary data.

### Electrophysiology

Nerve conduction studies and needle electromyography were performed in the probands and in additional affected relatives from the UK and Irish families.

### Genetic studies

Genetic testing was performed in the Bristol Genetics Laboratory, using the UK Genetic Testing Network approved approach; a multi-gene panel assay utilising next generation sequencing (NGS). Genomic DNA was extracted from the peripheral white blood cells obtained from probands of all families according to standard methods. Enrichment was performed using a custom-designed 330 kb SureSelect capture (Agilent Technologies) targeting 56 genes associated with inherited peripheral neuropathy (IPN). Libraries were prepared from genomic DNA according to the manufacturer’s protocol and sequenced on an Illumina MiSeq (2 × 150 bp). For data analysis and filtering, a bespoke open-source pipeline using BWA and GATK was used to align data to the reference human genome (UCSC hg19). Variant classification was based on Association for Clinical Genetic Science (ACGS) Practice Guidelines (2013). Candidate pathogenic variants were confirmed by Sanger Sequencing using an Applied Biosystems 3730 analyser.

### Consent

All participants provided written informed consent to be involved in this study which was approved by local research ethics committees.

## Results

### Patients

Due to overlapping and variable clinical phenotypes the referrals were occasionally misleading indicating CMT1, hereditary sensory neuropathy or intermediate CMT phenotypes. Multi-gene panel testing was performed in a total of 22 patients with ‘mixed/intermediate’ CMT and out of the 8 positives 5 were proved to be an AARS mutation.

Lower limb predominant sensorimotor neuropathy was characteristic in UK family 1. All family members developed *pes cavus* and foot drop bilaterally leading to progressive walking difficulties at different adult ages. They all remained ambulant with orthotic support. Various degree of upper limb involvement was observed. The proband manifested with fluctuating weakness of finger and intrinsic hand muscles at disease onset with progressive hand muscles wasting at later stage.

Slowly progressive various degree of motor weakness affecting the lower extremities and subsequent walking problems were the leading symptoms in UK family 2. Tiptoe walking and feet deformities were present. Sensory changes were mild or absent and no upper limb involvement was associated, but we note that the index patient is only 20 years of age.

In UK family 3, the clinical presentation was very heterogeneous. The proband manifested with young adult onset asymmetric upper and lower limb involvement equally affecting motor and sensory functions. His father had stable feet deformities since childhood which progressed rapidly in his late 50s exclusively and symmetrically affecting his lower limbs causing predominantly motor and milder sensory loss. Development of bilateral foot drop and walking difficulties were common symptoms within the family.

A slowly progressive disease course and predominant lower limb motor loss was the common presentation in UK family 4. Initial symptoms presented either in symmetric or asymmetric fashion and striking clinical features were bilateral foot and toe drop with subsequent walking difficulties. Various degrees of weakness and wasting of intrinsic hand muscles with split hand formation were associated. Mild to moderate sensory changes were present.

Lower limb motor symptoms progressed slowly in Irish family 1 leading to different impact on their walking abilities with ambulatory loss in some family members. Sensory loss in lower extremities accompanied and upper limb involvement manifested in later ages.

Early onset slowly progressive and predominantly motor impairment affecting both upper and lower extremities was observed in Irish family 2. Toe walking and bilateral foot drop were common features. Associated milder sensory changes became apparent at later ages. Wasting and weakness of intrinsic hand muscles resulted in split hand deformity (Fig. 1; Table 2).

**Table 2:** Genetic and clinical characteristics of patients with *AARS*-related neuropathy in the UK/Irish cohort

Family/patient/age/sex	Origin	Nucleotide/amino acid change	Age onset	Clinical course	First sign	Clinical signs						Mobility/orthotics	Nerve conduction study	
						Distal lower limb			Distal upper limb				Motor NCV (m/s)	Motor/sensory
						Motor	Sensory	Deformity	Motor	Sensory	Deformity			
F1/PIII.1/50 years/M	UK	c.986G>A/p.Arg329His	30 years	Rapid fluctuant Symmetric LL Asymmetric UL	UL	++	++	Bilateral *pes cavus* Bilateral foot drop	++	++	R>L mild split hand	Walking difficulties/ Walking stick Bilateral hand splint	m 38–50 p absent	MS
F1/PII.4/77 years/M	UK	c.986G>A/p.Arg329His	<10 years	Slow progress Symmetric	LL	+++	++	Bilateral *pes cavus* Bilateral foot drop Bilateral toe drop	+	++	None	Walking difficulties/ Bilateral AFOs Crutches	intermed.	MS
F1/PII.6/70 years/M	UK	c.986G>A/p.Arg329His	53 years	Slow progress Symmetric	LL	++	++	Bilateral foot drop	None	None	None	walking difficulties/ bilateral AFOs	intermed.	MS
F2/PIII.1/20 years/M	UK	c.986G>A/p.Arg329His	12 years	Slow progress Symmetric	LL	+++	+	Bilateral tight Achilles *pes cavo-equinus*	None	None	None	Tiptoe walking / Insoles	m 40 p 24	MS
F3/PIII.1/32 years/M	UK	c.986G>A/p.Arg329His	28 years	Rapid progress Asymmetric	UL LL	++	++	Bilateral *pes cavus*	++	++	None	Walking difficulties/ AFO	m 43 p 34	MS
F3/PII.1/59 years/M	UK	c.986G>A/p.Arg329His	birth	Slow progress Symmetric	LL	+++	+	Bilateral tight Achilles Bilateral foot drop	None	None	None	Walking difficulties/ Feet surgeries	n/a	n/a
F4/PII.2/55 years/M	UK	c.986G>A/p.Arg329His	30 years	Slow progress Asymmetric	LL	+++	++	Bilateral foot drop Bilateral toe drop	+++	++	SPLIT hand	Walking difficulties/ Bilateral AFO Hand surgery	m 26 u 34	MS
F4/PII.5/49 years/M	UK	c.986G>A/p.Arg329His	18 years	Slow progress Symmetric	LL	+++	+	Bilateral *pes cavus* Bilateral foot drop	+	None	Mild split hand	Walking difficulties	intermed.	MS
F1/PIV.5/46 years/F	Ireland	c.986G>A/p.Arg329His	<10 years	Slow progress Symmetric	LL	++	++	Bilateral foot drop	+	None	None	Walking difficulties	m 39 u 44.9	MS
F1/PV.1/10 years/F	Ireland	c.986G>A/p.Arg329His	<10 years	Slow progress Symmetric	LL	+	+	Bilateral tight Achilles	None	None	None	Tiptoe walking	m 47.6 p 31.6	MS
F2/PII.3/37 years/M	Ireland	c.2063A>G/p.Glu688Gly	<10 years	Slow progress Symmetric	LL	+++	++	Bilateral tight Achilles Bilateral foot drop	++	++	Split hand	Tiptoe walking	u 28.7	MS
F2/PIII.1/6 years/M	Ireland	c.2063A>G/p.Glu688Gly	<1 years	Slow progress Symmetric	LL	++	n/a	Bilateral tight Achilles Bilateral foot drop	+	n/a	Mild split hand	Tiptoe walking bilateral AFOs	m 36.8 p 27.6	MS
F2/PIII.2/5 years/F	Ireland	c.2063A>G/p.Glu688Gly	<1 years	Slow progress Symmetric	LL	+	n/a	Bilateral tight Achilles Bilateral foot drop	+	n/a	Mild split hand	Tiptoe walking bilateral AFOs	m 29.3	MS

*F* family, *P* patient, *M* male, *F* female, *UL* upper limb, *LL* lower limb, *+* mild, *++* moderate, *+++* severe, *AFO* ankle foot orthesis, *n/a* not available, *m/s* metre per second, m median nerve, *p* peroneal nerve, *u* ulnar nerve, *intermed.* intermediate, *MS* motor and sensory

### Electrophysiology

A series of nerve conduction studies was performed in the proband (III.1) of UK family 1. Initially the multifocal patchy demyelinating changes with conduction block leading to the dispersion of the motor neuronal conduction velocities were more suggestive of CIDP, but CSF protein was normal and the patient did not improve on immunosuppressive therapy. Previous recordings indicated intermediate motor neuropathy both demyelinating and axonal in nature with multifocal patchy characteristics and severe sensory polyneuropathy. Family members (II.4 and II.6) had intermediate nerve conduction velocities indicating both demyelinating and axonal features. The proband (III.1) of UK family 2 showed both demyelinating and axonal sensorimotor neuropathy with nerve conduction velocities in the intermediate range. The index patient (III.1) of UK family 3 had intermediate nerve conduction studies underlying his progressive asymmetrical distal sensorimotor neuropathy symptoms. The proband (II.2) of UK family 4 had motor conduction velocities in the demyelinating range consistent with demyelinating CMT (CMT1). Nerve conduction studies in his father (I.1) also showed profound demyelinating neuropathy with significant axonal loss. Testing of both his affected brothers (II.3 and II.5) revealed severe predominantly axonal peripheral sensorimotor neuropathy with reduced conduction velocities. Neurophysiology in the Irish families confirmed length-dependent motor and sensory neuropathy with intermediate conduction velocities (Supplementary Table).

### Genetic studies

Clinical characteristics and electrophysiology findings were directive in the candidate gene testing. Mutations in genes involved in axonal pathology (*MPZ*, *MFN2*, *NEFL*, *GDAP1*) and common demyelinating genes (*PMP22*, *MPZ*) were excluded in all patients. Upper limb involvement at disease onset and split hand deformity indicated exclusion of mutations in the *GARS* gene. Early onset lower extremity predominant motor symptoms jointly with Achilles contracture urged us to exclude the recently reported bicaudal D homolog 2 (BICD2)-related neuropathy. Prominent sensory manifestation in some patients indicated screening for *SPTLC1* mutations.

**Fig. 2:**
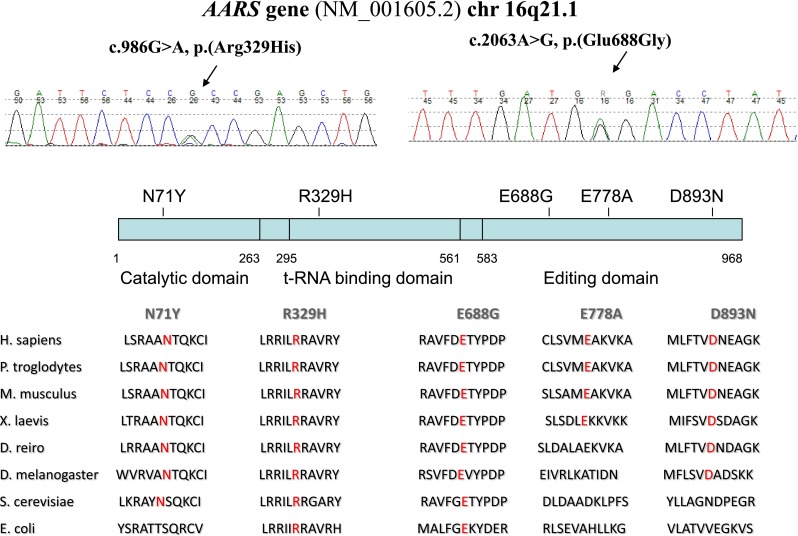
Sequenced c.986G>A missense mutation in *AARS* in affected UK and Irish index patients. Illustrated positions of *AARS* mutations and conservation of affected residues across species

With the use of IPN 56 gene panel assay we identified the previously described pathogenic variant in exon 8 of the *AARS* gene (c.986G>A, p.Arg329His) in all 4 UK and in Irish family 1. We identified another *AARS* variant (c.2063A>G, p.Glu688Gly) which to date has not been reported in the literature, as the cause of the dominant sensorimotor neuropathy manifesting in the second family originated from Ireland. This variant is not recorded in dbSNP, 1000 genomes, Exome Variant Server or Exome Aggregation Consortium. Alignment of protein sequences from multiple species supported that glutamic acid 688 is highly conserved among all species from *E.coli* to *H.sapiens*. *In silico* prediction tools indicate this missense change as likely deleterious (SIFT: deleterious; Polyphen2: probably pathogenic; Mutation Taster disease causing). The variant segregated with disease in this family supporting pathogenicity (Fig. 2).

## Discussion

Aminoacyl-tRNA synthetases (ARSs) catalyse a two-step aminoacylation reaction by binding and activating amino acids and conjugate them with their cognate tRNA molecules [3]. Their essential role in keeping the fidelity of the genetic code during protein translation explains why ARSs are ubiquitous and highly conserved amongst species [5]. Several hypotheses have been proposed in the mechanisms of ARS-related CMT pathology. Most mutations are missense amino acid substitutions leading to dominant CMT phenotypes [3, 7]. In vitro aminoacylation assays indicate both quantitative error via impaired enzyme activation, and qualitative defects by non-cognate bindings. Yeast viability assays investigated in vivo functional consequences of the ARS mutations and support a loss of function effect. Protein localisation studies show altered distribution of some of the ARS proteins in cultured neurons suggesting spatially inappropriate protein synthesis [3, 7, 9].

Alanyl–aminoacyl-tRNA synthetase (*AARS*) consists of 21 exons and encodes 968 amino acids. From the N-terminal to the C-terminal it has an aminoacylation or catalytic domain (AD), helical or tRNA-binding domain (HD) and editing domain (ED). Unlike as in other ARSs, the acceptor arm of tRNA^Ala^ can be directly recognised by the catalytic site of AARS. The editing domain was evolutionarily integrated in the AARS with the aim of eliminating mischarged tRNA^Ala^. Therefore, AARS has highly conserved sequence in all of its domains across species from *E. coli* to *H. sapiens* [5, 15].

In the literature, *AARS* mutations have been reported affecting all three domains. The p.(Asn71Tyr) *AARS* mutation, which was described in a Taiwanese pedigree, is located in the aminoacylation domain and both in vitro and in vivo assays revealed impaired aminoacylation activity result in reduced charging capacity [6, 7]. The recurrent, p.(Arg329His) mutation located in the middle helical domain of AARS has been largely investigated. Haplotype analysis in 2 French and 1 Australian family with Arg329His AARS mutation demonstrated a different founder for all three families. A methylation-mediated process gives rise to this recurrent mutation which was also found to associate with impaired enzyme activity. *AARS* exon 8, which contains the p.(Arg329His) variant, has a highly methylated CpG site and considered to be a mutational hot spot [7]. In the cohort reported here, this was the most common *AARS* mutation affecting one Irish and four UK families. A limited haplotype analysis of four polymorphic variants in the proximity of the causative *AARS* mutation showed identical haplotype for all four North UK families, and close similarity was suggested (3/4 identical variants) in the Irish family. These data imply that the p.(Arg329His) mutation is potentially a founder mutation in the five reported families (data not shown). The phenotypic manifestation was rather heterogeneous within the families despite the same genotypic background. However, lower limb predominance was characteristic in all families; significant upper limb involvement with split hand (selective atrophy of thenar muscles) could not be ignored in some patients similar to GARS-related pathology. Acute or sub-acute episodes of worsening, resembling acquired neuropathies were also reported. Compared to the previously reported pedigrees, nerve conduction studies in our families showed greater demyelination process accompanying the always present axonal dysfunction. Sensory responses were frequently severely impaired along with occasional clinically significant sensory loss. Similar to the French pedigree, we observed asymmetric distribution of the symptoms in some of the patients, but in contrast to the Australian cohort, sensorineural deafness was not present in either of the families **(**Tables 1, 2). There have been two different phenotypes described so far in relation to the *AARS* editing domain mutations. The p.(Glu778Ala) variant manifesting in an Australian family with dominant rippling muscle disease, did not involve an evolutionarily conserved amino acid within the AARS editing domain and impairment in the editing capacity also could not be proven [7]. In a Chinese pedigree, the p.(Asp893Asn) mutation led to a dominant pure motor pathology causing dHMN. It was found to reside in a highly conserved sequence of the C-terminal editing domain and prediction programs indicated deleterious pathogenicity [15]. In our cohort, we identified a novel *AARS* variant which also locates to the AARS editing domain. The clinical phenotype related to p.(Glu688Gly) is of an early onset length-dependent motor and sensory neuropathy. Neurophysiology, similar to our families with the p.Arg329His mutation, showed intermediate conduction velocities. In the proband, there was also more marked involvement of FDIO and APB than ADM, suggesting a split hand phenotype. Functional studies would be necessary to further analyse the pathogenicity of this variant and its impact on the editing capacity of the enzyme.

Nothing indicates better the variability of *AARS*-related pathology than an utmost recent paper, published during the revision of our manuscript, which describes a novel heterozygous missense c.304G>C (p.Gly102Arg) *AARS* variant manifesting with a novel myeloneuropathy phenotype in a large family [8]. This warrants that genetic screening toward *AARS* and other aminoacyl-tRNA synthetase mutations might be always considered in axonal neuropathology.

## Conclusions

*AARS*-related neuropathy was identified in a cohort of dominant UK and Irish families by multi-gene panel approach. Our cohort supports that the p.(Arg329His) *AARS* variant is a recurrent mutation presenting worldwide. The associated phenotypic spectrum is heterogeneous and may cause difficulties in achieving diagnosis based only on clinical examination, underlying the valuable contribution of next generation sequencing.

## Electronic supplementary material

Supplementary material 1 (DOCX 26 kb)
