# Supplementary material for: Genotype/phenotype correlations in AARS-related neuropathy in a cohort of patients from the United Kingdom and Ireland
Source: J Neurol. 2015 Jun 2;262(8):1899–908. doi: 10.1007/s00415-015-7778-4 (PMC4539360; doi:10.1007/s00415-015-7778-4)
Supplement: Supplementary file 1 — Supplementary material 1 (DOCX 26 kb) [file 415_2015_7778_MOESM1_ESM.docx]

**Genotype/phenotype correlations in AARS-related neuropathy in a cohort of patients from the United Kingdom and Ireland**

Boglarka Bansagi^(1)*^, Thalia Antoniadi^(2)*^, Sarah Burton-Jones^(2)^, Sinead M Murphy^(3)^, John McHugh^(4)^, Michael Alexander^(4)^, Richard Wells^(2)^, Joanna Davies^(2)^, David Hilton-Jones^(5)^, Hanns Lochmüller^(6)^, Patrick Chinnery^(1)^, Rita Horvath^(1)^

(1) MRC Centre for Neuromuscular Diseases, Institute of Genetic Medicine, Newcastle University, Newcastle upon Tyne, UK

(2) Bristol Genetics Laboratory, Pathology Sciences, North Bristol NHS Trust, Southmead Hospital, Bristol, UK

(3) Department of Neurology, Adelaide & Meath Hospitals Incorporating the National Children’s Hospital, Tallaght, Dublin 24 and Academic Unit of Neurology, Trinity College Dublin, Ireland

(4) Department of Neurophysiology, Adelaide & Meath Hospitals Incorporating the National Children’s Hospital, Tallaght, Dublin 24, Ireland

(5) Department of Neurology, West Wing, John Radcliffe Hospital, Oxford, UK

(6) John Walton Muscular Dystrophy Research Centre, MRC Centre for Neuromuscular Diseases, Institute of Genetic Medicine, Newcastle University Newcastle upon Tyne, UK

*BB and TA contributed equally to this work.

**Corresponding Author**

Rita Horvath MD PhD

Institute of Genetic Medicine, Newcastle University

Newcastle upon Tyne, NE1 3BZ United Kingdom

Tel: +44 191 2418855, Fax: +44 191 2418666

Email: [rita.horvath@ncl.ac.uk](mailto:rita.horvath@ncl.ac.uk)

**Supplementary data**

**UK Family 1**

The 50 year old male proband (III.1) **(Figure 1.B)** from a three generational dominant North-British family presented with sudden onset of symptoms, over a period of weeks, around the age of 30. He developed symmetric distal weakness in his upper limbs impairing his dexterity. He experienced decreased grip strength and numbness in his hands. Later the clinical course became rather fluctuating with some improvement with regard to the upper limb involvement but deteriorating with painful dysaesthesia and distal muscle weakness in the lower extremities. His gait was impaired, with frequent falls. Aged 33 he manifested with acute asymmetrical loss of power and sensation on the right side along with speech difficulties, resembling of an acute ischaemic cerebral attack (the clear background has remained uncertain despite thoroughly performed investigations including neuroimaging). Later on, his clinical manifestations were compatible with a lower limb predominant sensorimotor neuropathy. Nerve conduction studies suggested a chronic inflammatory demyelinating neuropathy (CIDP) but he was unresponsive to therapy trials of steroids and IVIG.

His recent neurology examination revealed a right sided predominant moderate weakness in his finger and hand muscles at MRC grade 4/5. He was provided with bilateral hand splints to aid his manual skills. There was a more severe weakness present in the ankle plantar- and dorsiflexion at MRC grade 3/5 and he was noted to have bilateral pes cavus and severe foot drop. Sensory loss for pinprick and vibration was indicated below mid calves and wrists. He was walking with stepping gait with poor balance requiring the aid of a walking stick. No cranial nerve involvement, pyramidal signs and cerebellar symptoms was observed and his cognition was preserved.

The 77 year old father of the proband (II.4) **(Figure 1.B)** presented with a very slowly progressive neuropathy with no fluctuations since early childhood and causing problems only in his late 60’s. However he had history of foot surgeries dated back to his childhood. In his 60’s he started limping which worsened by exercise, with no significant peripheral vascular pathology. He manifested with constant pain in his lower limbs with a distribution characteristics for lumboischialgia but spinal imaging was unremarkable. He progressed rapidly after the age of 70 losing his balance and falling frequently. Recently he started to manifest with symptoms of parkinsonism. On examination he had distal predominant muscle wasting and weakness in the legs. His muscle strength in the feet was MRC grade 1/5 and his toes were not moving at all. He had marked pes cavus and bilateral severe foot drop. There was hardly any upper limb involvement apart from a very mild atrophy and weakness in the hand muscles at an MRC grade 4+/5. Deep tendon reflexes were entirely absent. Pinprick sensation was impaired below his wrists and ankles while sensation of vibration was lost below his knees and elbows. He was wearing bilateral splints and required crutches to aid his walk.

Two of his 6 brothers (II.1 and II.6) and his mother (I.1) were also affected by peripheral neuropathy. His brother (II.6) developed distal sensory and motor loss with bilateral foot drop at the age of 53 but he remained ambulant using splints. **(Figure 1.A) (Table 2)**

**UK Family 2**

The proband (III.1) is a 20 year old young man from the North East of England, who presented with a slowly progressive course of the disease with an age onset at 12 years. He developed tightness in his Achilles tendons with subsequent tiptoeing and clumsiness. He progressed further manifesting with distal muscle weakness jointly with pain affecting his lower extremities. His foot deformity was corrected by osteotomy and tendon transfer on the right side. On examination marked muscle wasting and weakness was present symmetrically in his legs and he had bilateral cavo-equinus foot deformity. Deep tendon reflexes could not be elicited. He was noted to have evolving mild pinprick sensory loss below his ankles. He was ambulant using insoles walking with stepping gait. There were no evidence of upper limb involvement and the rest of his neurology assessment was also normal.

His family history indicated autosomal dominant inherited neuropathy. His father (II.2) showed a similar but rather moderate disease course and he remained independently ambulant with no need of orthotic aids. His paternal aunt (II.1) and paternal grandmother (I.1) were also reported to be affected and the latter required walking aid to remain ambulant. **(Figure 1.A) (Table 2)**

**UK Family 3**

The 32 year old proband (III.1) of this North Eastern UK family had a longstanding history of ankylosing spondylitis treated with combined anti-inflammatory and immunotherapy. He required subsequent bilateral hip replacement. He was reported to be clumsy as a child and aged 28 he developed asymmetric weakness in the left side of his body associated with intermittent pins and needles and numbness. He experienced difficulties typing and writing with his left hand due to the decreased grip strength and weakness in his finger muscles. He reported being generally fatigued, feeling tired and lethargic. There was no evidence of inflammatory demyelination by performing neuroimaging and CSF analysis. He was assessed with left sided predominant muscle wasting and weakness in his distal extremities. Moderate weakness and atrophy affected his finger muscles at an MRC grade 3-4/5. His ankle dorsi and plantarflexion was MRC grade 4/5 and he had bilateral pes cavus. He was areflexic. Light touch and pinprick impairment was noted over his entire left hand. His walking is supported with orthotic aid.

The family history was strongly suggestive of dominantly inherited peripheral neuropathy. His father (II.1) **(Figure 1B)** had feet deformities at birth which required surgical intervention at around the age of 1. Despite he was tiptoe walking over his childhood he trained to be a football coach and he was actively playing football until his late 50’s. He deteriorated rapidly, over a year manifesting with distal lower limb wasting and weakness accompanied by significant pain in his ankle joints. He developed bilateral severe foot drop with walking difficulties. On assessment dorsiflexion of his ankles was at MRC grade 3/5 and plantar flexion was 1/5 bilaterally. Lower limb reflexes were absent with no long tract signs. Muscle bulk and muscle strength in his upper limbs were preserved and upper limb reflexes were present. He had Dupuytren’s contracture on his right hand. Mild hyperaesthesia was noted in his feet for pin prick test.

His paternal grandfather (I.1) is 82 years old and has bilateral foot drop and requires splints to remain ambulant. His paternal uncle (II.2) underwent surgical intervention for some ankle problems and his two sons are investigated for orthopaedic issues. **(Figure 1A) (Table 2)**

**UK Family 4**

The 55 year old proband (II.2) developed severe low back pain and right sided sciatica around the age of 22 and a myelogram showed prolapsed disc. Couple years later in his 30’s his right leg became thinner than the left and he started walking with dropped foot. He had progressive weakness in his both legs, the right remaining more severely affected. His lower limbs were wasting from the lower thighs distally. His striking clinical feature at presentation was the appearance of dropped toes. He experienced some numbness in his feet from ankles downwards and he could not appreciate vibration sensation below his knees. Some time later he noted weakness and wasting of the small muscles in his hands and there was marked weakness noted in finger abduction. Superficial sensation was impaired distally over his fingers. His condition has slowly deteriorated over the years. Aged 40 he manifested with walking difficulties, increasing falls and feelings of instability despite using a single walking stick. He was provided with ankle orthotics and fusion surgery was planned. He displayed substantial difficulties with his hands and due to impaired hand function he underwent tendon transfer surgery to achieve some thumb function.

There is a dominant family history with all affected relatives origin from Northern England. His father (I.1) had progressive walking difficulties with poor balance control since his 40’s and he was eventually diagnosed with chronic demyelinating neuropathy at the age 60. Two of his four brothers (II.3 and II.5) were diagnosed with intermediate CMT. They had a very similar disease course and onset of symptoms at their late teens with progressive ankle instability and frequent ankle sprains. They presented with progressive distal amyotrophy and bilateral pes cavus from their 20’s. They progressed slowly manifesting with muscle weakness in the lower extremities, areflexia and bilateral foot drop which resulted in significant walking difficulties in their middle ages. It was associated with a mild sensory impairment in their feet. The younger of them (II.5) was also noted with bilateral wasting of the first dorsal interossei with no subsequent impairment in his hand function. The next generation has not yet presented with functional problems although the oldest son of patient II.2 has a tendency for his big toes to drop and the youngest son of patient II.3 has overriding toes. **(Figure 1A) (Table 2)**

**Irish Family 1**

The 46 year old proband (IV.5) was always poor at sports in the first decade and had recurrent patellar dislocations in childhood. Her walking and balance slowly deteriorated and she became aware of reduced sensation in the feet in her mid-30s. In her 40s she developed some difficulty with writing and typing. On examination there was mild ptosis and a high arched palate. She walked with mild bilateral foot drop and a slight waddle. She had weakness to grade 4 in intrinsic hand muscles, grade 4 hip flexion and grade 2 ankle dorsiflexion with grade 4 plantarflexion. She was areflexic. Pin sensation was reduced to ankles, vibration reduced to costal margins. CMTNS2 score was 15/36 indicating moderate severity CMT. [10]

There was an autosomal dominant family history **( Figure 1A)**. Her daughter (V.1) toe walked and was clumsy in the first decade. At 7 years she had evidence of mild proximal and distal lower limb weakness, absent ankle reflexes and diminished fine touch distally in the feet. The proband’s father (III.5) had longstanding schizophrenia but a stamping gait had been reported for many years previously. He developed slow deterioration in gait with wasting and weakness of the hands and distal legs. He developed swallowing difficulties at 70, requiring PEG tube insertion. He became wheelchair bound at 76. Several other family members became wheelchair bound in later life (I.2, II.1, II.2, II.5). **(Figure 1A) (Table 2)**

**Irish Family 2**

The 37 year old proband (II.3) was clumsy at walking in the first decade and was always slowest in races. He would turn easily on his ankles and fell frequently. He started to notice difficulty with fine movements of his hands in his teens. Symptoms progressed gradually. He now has a tremor and difficulty doing up buttons. He finds it hard to stand still and falls easily. On examination, he toe walks with bilateral foot drop. The Achilles tendons are tight bilaterally. There was wasting from the mid forearm and from the knees distally. He had weakness of intrinsic hand muscles; abductor pollicis brevis (APB) grade 1, first dorsal interosseous (FDIO) 4- and abductor digiti minimi (ADM) grade 4. Ankle dorsiflexion and plantarflexion were grade 1. He was areflexic. Sensation to pin was reduced to above the wrists and proximal shin. Vibration was reduced to costal margin bilaterally. Proprioception was normal. CMTES2 score was 17/28.[7]

There was a dominant family history, both his children being affected **(Figure 1A)**. His 6-year-old son (III.1) toe walked from onset and fell frequently. He had a poor pencil grip. He wore AFOs from the age of 5. On examination he walked with bilateral foot drop. He had mild weakness of intrinsic hand muscles, APB weaker than FDIO, and of ankle dorsiflexion. He was areflexic throughout. The proband’s 5-year-old daughter (III.2) had delayed motor and developmental milestones. She toe walks, falls frequently and wears boots for ankle support. She attends a special needs school. She walks with bilateral foot drop. There was mild APB and ankle dorsiflexion weakness. Reflexes were absent apart from at triceps and knees. Sensory testing was unreliable in the children. **(Figure 1A) (Table 2)**

**Supplementary Table:** Summary of neurophysiology results in UK / Irish families

| **Patient** | **Age/ Sex** | **Median SAP (uV)** | **Median SNCV (m/s)** | **Ulnar SAP (uV)** | **Ulnar SNCV (m/s)** | **Radial SAP (uV)** | **Radial SNCV (m/s)** | **Sural SAP (uV)** | **Sural SNCV (m/s)** | **Median CMAP (mV)** | **Median MNCV (m/s)** | **Ulnar CMAP FDIO (mV)** | **Ulnar MNCV**  **FDIO (m/s)** | **Ulnar CMAP ADM (mV)** | **Ulnar**  **MNCV**  **ADM**  **(m/s)** | **Peroneal CMAP (mV)** | **Peroneal MNCV (m/s)** | **Tibial CMAP (mV)** | **Tibial MNCV (m/s)** |
| --- | --- | --- | --- | --- | --- | --- | --- | --- | --- | --- | --- | --- | --- | --- | --- | --- | --- | --- | --- |
| **UK families** |  |  |  |  |  |  |  |  |  |  |  |  |  |  |  |  |  |  |  |
| F1/PIII.1 | 50y M | NR | NR | NR | NR | NR | NR | NR | NR | 2 | 27 | n/a | n/a | 9.3 | 46 | NR | NR | NR | NR |
| F2/PIII.1 | 20y M | 3 | 31 | 1 | 30 | 3 | 35 | NR | NR | 14.4 | 40 | n/a | n/a | 11 | 44 | 3 | 24 | 3.6 | 31 |
| F3/PIII.1 | 32y M | 1 | 50 | NR | NR | 8 | 45 | NR | NR | 18 | 43 | n/a | n/a | 18 | 47 | NR | NR | 0.4 | 34 |
| F4/PII.2 | 55y M | NR | NR | n/a | n/a | n/a | n/a | n/a | n/a | 2.5 | 26 | n/a | n/a | 3.3 | 34 | n/a | n/a | n/a | n/a |
| **Irish families** |  |  |  |  |  |  |  |  |  |  |  |  |  |  |  |  |  |  |  |
| F1/IV.5 | 46 F | 0.4 | 33.3 | n/a | n/a | 1.2 | 53.8 | NR | NR | 4.5 | 39 | 7.1 | 44.9 | n/a | n/a | NR | NR | 2.6 | n/a |
| F1/V.1 | 7 F | 5.8 | 34.8 | 4.7 | 36 | 11.6 | n/a | n/a | n/a | 9.7 | 47.6 | 17.75 | 46.3 | n/a | n/a | 2.44 | 31.6 | 5.81 | 39.7 |
| F2/II.3 | 37 M | NR | NR | NR | NR | NR | NR | NR | NR | NR | NR | 0.11 | 28.7 | 3.4 | 28.5 | NR | NR | NR | NR |
| F2/III.1 | 6 M | NR | NR | n/a | n/a | NR | NR | NR | NR | 3.2 | 36.8 | n/a | n/a | 7.6 | 44 | 1.35 | 27.6 | 10.2 | 38.1 |
| F2/III.2 | 5 F | NR | NR | n/a | n/a | n/a | n/a | n/a | n/a | 3.6 | 29.3 | n/a | n/a | n/a | n/a | n/a | n/a | n/a | n/a |

F, family; P, patient; y, year; M, male; F, female; SAP, sensory action potential; SNCV, sensory nerve conduction velocity; CMAP, compound motor action potential; MNCV, motor nerve conduction velocity; FDIO, first dorsal interosseous; ADM, abductor digiti minimi; NR, not recordable; n/a, not available
